# Identification of latent neosporosis in sheep in Tehran, Iran by polymerase chain reaction using primers specific for the *Nc-5* gene

**DOI:** 10.4102/ojvr.v83i1.1058

**Published:** 2016-08-11

**Authors:** Mohsen Arbabi, Amir Abdoli, Abdolhossein Dalimi, Majid Pirestani

**Affiliations:** 1 Department of Parasitology, Kashan University of Medical Sciences, Iran; 2 Department of Parasitology, Tarbiat Modares University, Iran

## Abstract

Little is known about latent infection and molecular characterisation of *Neospora caninum* in sheep (*Ovis aries*). In this study, 330 sheep samples (180 hearts and 150 brains) were analysed for *N. caninum* DNA by nested polymerase chain reaction (PCR) targeting the *Nc-5* gene. *Neospora caninum* DNA was detected in 3.9% (13/330) of sheep samples. The parasite’s DNA was detected in 6.7% of heart samples (12/180) and 0.7% (1/150) of brain samples. No clinical signs were recorded from infected or uninfected animals. Sequencing of the genomic DNA revealed 96% – 99% similarity with each other and 95.15% – 100% similarity with *N. caninum* sequences deposited in GenBank. To our knowledge, this is the first report on the use of PCR to identify latent neosporosis in sheep in Iran. The results of this study have the potential to contribute to our understanding of the role of *N. caninum*-infected sheep in the epidemiology of neosporosis.

## Introduction

*Neospora caninum* is a worldwide protozoan having a variety of animal hosts (Dubey & Schares 2011; Dubey, Schares & Ortega-Mora 2007). Domestic and wild canids are definitive, whereas different bird and mammalian species (such as cattle, water buffalo, and sheep) serve as intermediate hosts (Dubey & Schares 2011). Abortion, especially in dairy cattle, is one of the major consequences of neosporosis in animal husbandry (Almeria & López-Gatius 2013) that lead to significant economic losses (Reichel *et al*. 2013). Moreover, ovine abortion and reproductive failure due to neosporosis have been reported in several studies (Dubey & Lindsay 1990; Howe *et al*. 2008, 2012; Jolley *et al*. 1999; Moreno *et al*. 2012; Pena *et al*. 2007). In different studies, antibodies to *N. caninum* have been detected in 1.1% – 8.3% of sheep in the west of Iran (Ezatpour *et al*. 2015; Gharekhani & Heidari 2014), 27.7% in Pakistan (Nasir *et al*. 2012), 2.1% in Turkey (Gökçe *et al*. 2015), 10.3% in China (Liu *et al*. 2015), 16.8% in Greece (Anastasia *et al*. 2013), 3% in Argentina (Hecker *et al*. 2013), and 13.1% in south-eastern Brazil (Da Silva Andrade *et al*. 2012). However, there is little information describing the detection of nucleic acids resulting from latent neosporosis in sheep.

Until now, different genes such as internal transcribed spacer sequences, 18S-like ribosomal DNA (small-subunit rDNA), and *Nc-5* genes have been used for molecular diagnosis of neosporosis (reviewed by Al-Qassab, Reichel & Ellis [2010]; Goodswen, Kennedy & Ellis [2013]). However, studies have indicated that the *Nc-5* gene is one of the most highly sensitive and specific for the detection of neosporosis (Almerıa *et al*. 2002; Dubey *et al*. 2014; Hughes *et al*. 2006; Kaufmann *et al*. 1996; Paula *et al*. 2004; Yamage, Flechtner & Gottstein 1996) because it is repeated in the *N. caninum* sequence (Al-Qassab *et al*. 2010). Hence, the main objective of this study was to investigate detection and molecular characterisation of latent neosporosis in sheep (*Ovis aries*) in Tehran, Iran, by polymerase chain reaction (PCR) using primers specific for the *Nc-5* gene.

## Materials and methods

### Animals and study area

A total of 330 samples from healthy slaughtered sheep (180 hearts and 150 brains) were purchased from an abattoir in Vavan (located in the vicinity of Tehran) from April to September 2014. The animals tested originated from different counties (Eslamshahr, Shahriar, Robatkarim), all of which are located between 50 km and 200 km from Tehran. These locations have hot summers and moderate winters. No clinical signs such as fever, lymphadenitis, nasal and ocular discharges, or jaundice were recorded in any of the animals before slaughter.

### DNA extraction

The whole brain and heart of each sheep were individually rinsed with distilled water, packaged, and refrigerated. Approximately 200 g – 250 g of different segments of brain and heart were homogenised with a pestle and mortar in liquid nitrogen, and DNA was extracted using a phenol–chloroform extraction method as described in our recent report (Abdoli *et al*. 2015). To prevent DNA cross-contamination, all materials that were used between different tissue samples were decontaminated with sodium hypochlorite solution (2.5%) and rinsed with distilled water. The concentration of DNA was determined by NanoDrop spectrophotometer (Thermo Fisher Scientific, Wilmington, DE, USA) for each sample. Overall, the DNA concentration ranged between 150 ng/μL and 200 ng/μL.

### Nested polymerase chain reaction

Nested PCR was conducted using specific primers for the *Nc-5* gene. The first round of PCR was conducted using a pair of *N. caninum*–specific primers, Np21plus (5’-CCCAGTGCGTCCAATCCTGTAAC-3’) and Np6plus (5’-CTCGCCAGTCCAACCTACGTCTTCT-3’) (Muller *et al*. 1996). Nested PCR was performed with the primers Np6 (5’-CAGTCAACCTACGTCTTCT-3’) and Np7 (5’-GGGTGAACCGAGGGAGTTG-3’) (Hughes *et al*. 2006). Each amplification was performed in 20-µL reaction mixtures containing 10 µL of 2x master mixes (DFS Master Mix, BIORON GmbH, Ludwigshafen, Germany), each of the respective primers (10 pmol for the first round reaction and 25 pmol for nested PCR), 7 µL of distilled water, and 1 µL of template DNA. One microlitre of the first round product was used as the template for nested PCR. For each reaction, a negative control (double distilled water) and a positive control (DNA extracted from the *Nc-5* strain of *N. caninum*) were included. Amplification was performed with initial denaturation for 5 minutes at 94 °C, followed by 40 cycles at 94 °C for 40 seconds (denaturation), annealing at 62 °C in the first round, and 56 °C in nested PCR for 40 seconds, extension at 72 °C for 40 seconds, and final extension at 72 °C for 10 minutes. PCR products were electrophoresed on a 1.5% agarose gel stained with safe stain (Sinaclon, Tehran, Iran) and visualised under ultraviolet trans-illumination.

### Nucleotide sequence analysis

Four positive PCR products (from the second reaction) were amplified with a master mix containing *Pfu* DNA polymerase (Thermo Fisher Scientific, Waltham, USA, cat. no. EP0501), extracted from the gel (Vivantis gel purification kit, Selangor Darul Ehsan, Malaysia) according to the manufacturer’s protocols. Then the products were sequenced in the forward and reverse directions by Sequetech (Mountain View, CA, USA) (Abdoli *et al*. 2015). The sequences were edited with BioEdit sequence alignment editor (Hall 1999), aligned with *Nc-5* partial sequences from other hosts by ClustalX2.12 (Larkin *et al*. 2007) and compared with sequences of *N. caninum* available in GenBank. Phylogenetic trees were inferred and evolutionary analyses were conducted using the Tamura three-parameter option of the neighbour-joining model with MEGA6 software (http://www.megasoftware.net/) (Tamura *et al*. 2013). The bootstrap scores were calculated for 1000 replicates (Tamura *et al*. 2013).

### Results

*Neospora caninum* DNA was detected in 13 out of 330 sheep samples (3.9%). The infection rates in the heart and brain samples were 6.7% (12/180) and 0.7% (1/150), respectively. Four nucleotide sequences of the *Nc-5* gene with a length of 227 bp (Figure 1) were submitted to the GenBank database (GenBank accession numbers KR106181, KR106182, KR106183, KR106184). The results demonstrated our sequences shared 96% – 99% similarity with each other (Figures 2 and 3) and 95% – 100% similarity with *N. caninum* deposited in GenBank (Appendix Figure 1). Phylogenetic trees showed intraspecific variations between our isolates and other *N. caninum* specimens deposited in GenBank (Figure 2). Analysis of our sequences showed 96.9% – 97.8% similarity with *N. caninum* isolated from sheep (DQ077661) in the UK and 96.9% – 99.1% similarity with *N. caninum* isolated from sparrows (*Passer domesticus*) in Iran. Interestingly, one of our samples (KR106181) showed 100% similarity with *N. caninum* isolated from wolves (*Canis lupus*) (KF649846) in the United States.

**FIGURE 1:**
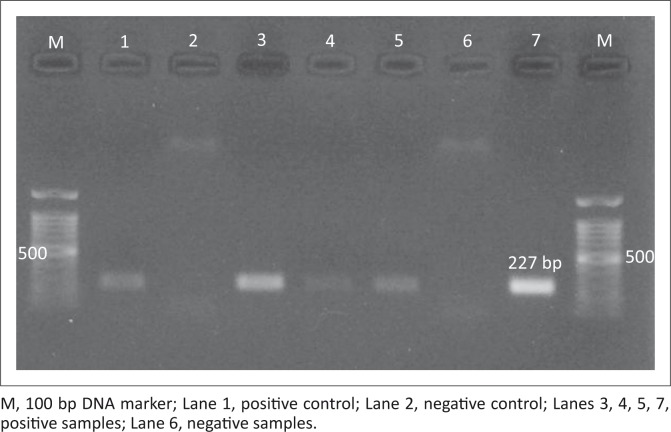
Polymerase chain reaction products of four *Neospora caninum* positive samples.

**FIGURE 2:**
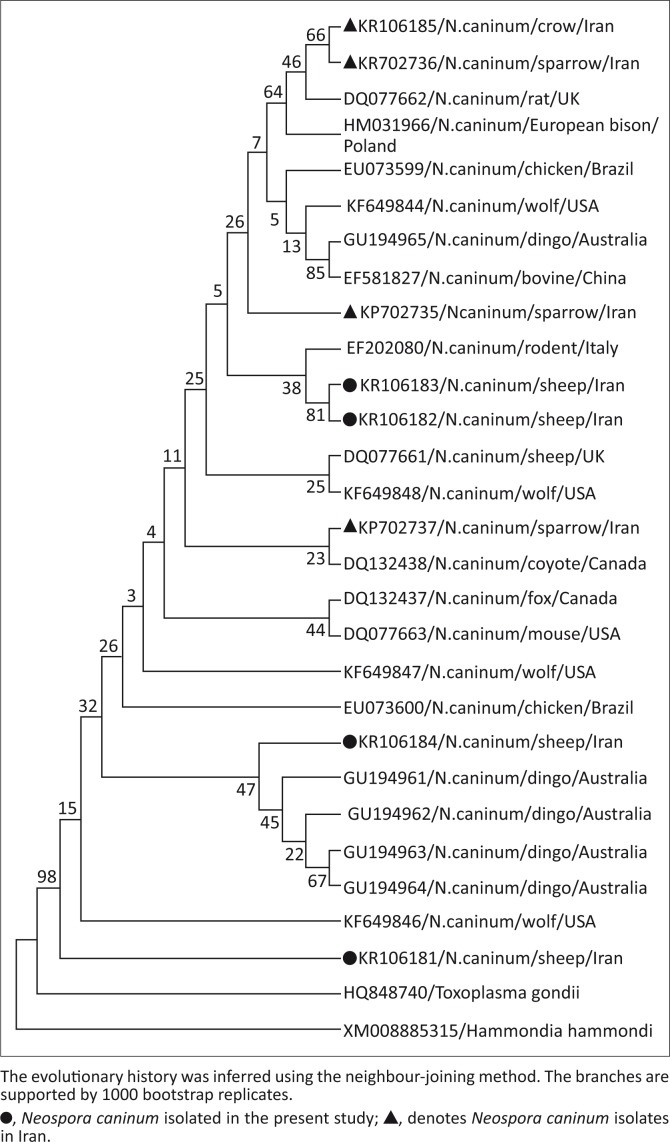
Phylogenetic relationships among *Neospora caninum* specimens based on a fragment of the *Nc-5* sequence.

**FIGURE 3:**
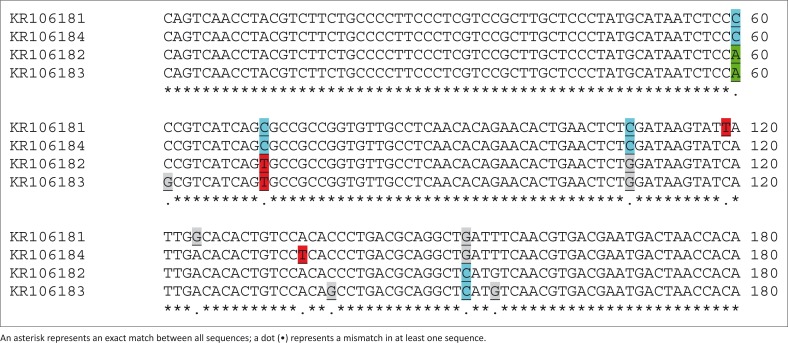
Partial sequences of the *Nc-5* gene from four isolates of *Neospora caninum* from sheep samples.

## Discussion

Although an association between ovine abortion and neosporosis has been reported in different studies (Dubey & Lindsay 1990; Howe *et al*. 2008, 2012; Jolley *et al*. 1999; Moreno *et al*. 2012; Pena *et al*. 2007), there is little information describing molecular detection of latent neosporosis in sheep. Here, we found a total infection rate of 3.9% (13/330) in our sheep samples. Interestingly, 12 out of 13 positive samples were detected in the hearts and one positive sample was diagnosed in the brain. In previous studies, the seroprevalence of *N. caninum* has been reported in a range of 1.1% – 8.3% of sheep from the west of Iran (Ezatpour *et al*. 2015; Gharekhani & Heidari 2014). Moreover, *N. caninum* DNA was detected in 8.5% (Asadpour *et al*. 2013) and 0.9% of aborted ovine fetuses in Iran (Sasani *et al*. 2013). Şuteu *et al*. detected *N. caninum* DNA in the diaphragm tissues of 2 out of 181 (1.1%) slaughtered goat kids in Romania (Şuteu *et al*. 2013). In the majority of studies, *N. caninum* was detected in brain samples from aborted or naturally infected sheep (Asadpour *et al*. 2013; Bishop *et al*. 2010; Dubey & Lindsay 1990; Sasani *et al*. 2013; Silva *et al*. 2009). In this regard, Silva and colleagues detected *N. caninum* DNA in 2 out of 102 slaughtered goats (1.92%) in Brazil. Interestingly, both positive samples were isolated from brain samples, whereas all heart and tongue samples were negative (Silva *et al*. 2009). Santos *et al*. (2010) detected *N. caninum* DNA in 5 out of 100 brain samples of beef cattle in Brazil, whereas none of the heart samples were positive (Santos *et al*. 2010). These results are dissimilar to our report, in which most of the positive samples were detected in the heart samples rather than in the brain samples (6.7% *versus* 0.7%). Our results also indicated that in sheep the heart is more susceptible to *N. caninum* infection than the brain.

Latent neosporosis can reactivate in conditions such as immunosuppression and pregnancy (Andrianarivo *et al*. 2005; Hemphill, Vonlaufen & Naguleswaran 2006; Magaña *et al*. 2015; Mazuz *et al*. 2016; Pabón *et al*. 2007; Rettigner *et al*. 2004). Latently infected animals are also a source of *N. caninum* infection for canine definitive hosts.

In the current study, we used the *Nc-5* gene for detection and phylogenetic analysis of *N. caninum*. This gene is repeated in the *N. caninum* sequence (Al-Qassab *et al*. 2010); hence, it is presented as a highly sensitive and specific gene for detection of neosporosis (Kaufmann *et al*. 1996; Yamage *et al*. 1996). The earlier study in this regard was conducted by Yamage *et al*. (1996), who compared the sensitivity and specificity of different primers for diagnosis *of N. caninum*. In this study, five forward (Np1, Np3, Np5, Np7, Np21) and four reverse (Np2, Np4, Np6, Np8) oligonucleotide primers that derived from the *Nc-5* genes were compared for the detection of neosporosis in experimentally infected mice. Among 19 combinations of forward and reverse primers, the Np21/Np6, Np7/Np6, and Np2l/Np4 primer pairs were able to detect at least 10 pg genomic DNA with a specific single band (Yamage *et al*. 1996). The *Nc-5* gene can also discriminate *N. caninum* from other related apicomplexan parasites (*Toxoplasma gondii* and *Sarcocystis* species) (Kaufmann *et al*. 1996). Thus, the *Nc-5* gene has been used as a highly sensitive and specific gene for detection of neosporosis (Almerıa *et al*. 2002; Dubey *et al*. 2014; Hughes *et al*. 2006; Paula *et al*. 2004; Yamage *et al*. 1996). Hence, we selected the *Nc-5* gene for sensitive and specific detection of neosporosis in the current study.

We also sequenced four positive samples for phylogenetic analysis. We found that our sequences displayed similarity levels of 96% – 99% with each other (Figure 3) and 95% – 100% with *N. caninum* sequences deposited in GenBank (Appendix Figure 1). In comparison with molecular diagnosis, few studies have been conducted on the phylogenetic analysis of *N. caninum* with the *Nc-5* gene (Auriemma *et al*. 2014; Čobádiová *et al*. 2013; Hughes *et al*. 2006). BLAST analyses indicated greater than 94% (Čobádiová *et al*. 2013), 96% (Auriemma *et al*. 2014), and 97% (Hughes *et al*. 2006) similarities between their sequences and other *N. caninum* sequences deposited in GenBank. It therefore seems that the *Nc-5* gene is not a suitable biomarker for phylogenetic analysis and discrimination of genetic diversity for *N. caninum*. Instead, this gene is rather a highly sensitive and specific biomarker for the diagnosis of neosporosis. The use of ribosomal DNA, ITS-1, and recently microsatellites have been recommended for discriminating between *N. caninum* isolates (Al-Qassab *et al*. 2010).

Taken together, the results of this study provide molecular and epidemiological information about latent *N. caninum* infection in sheep in Iran. It can be expected that in future these results will contribute to revealing the role of latent *N. caninum* infection in the epidemiology of neosporosis in sheep.

## Figures and Tables

**Figure 1-A1:**
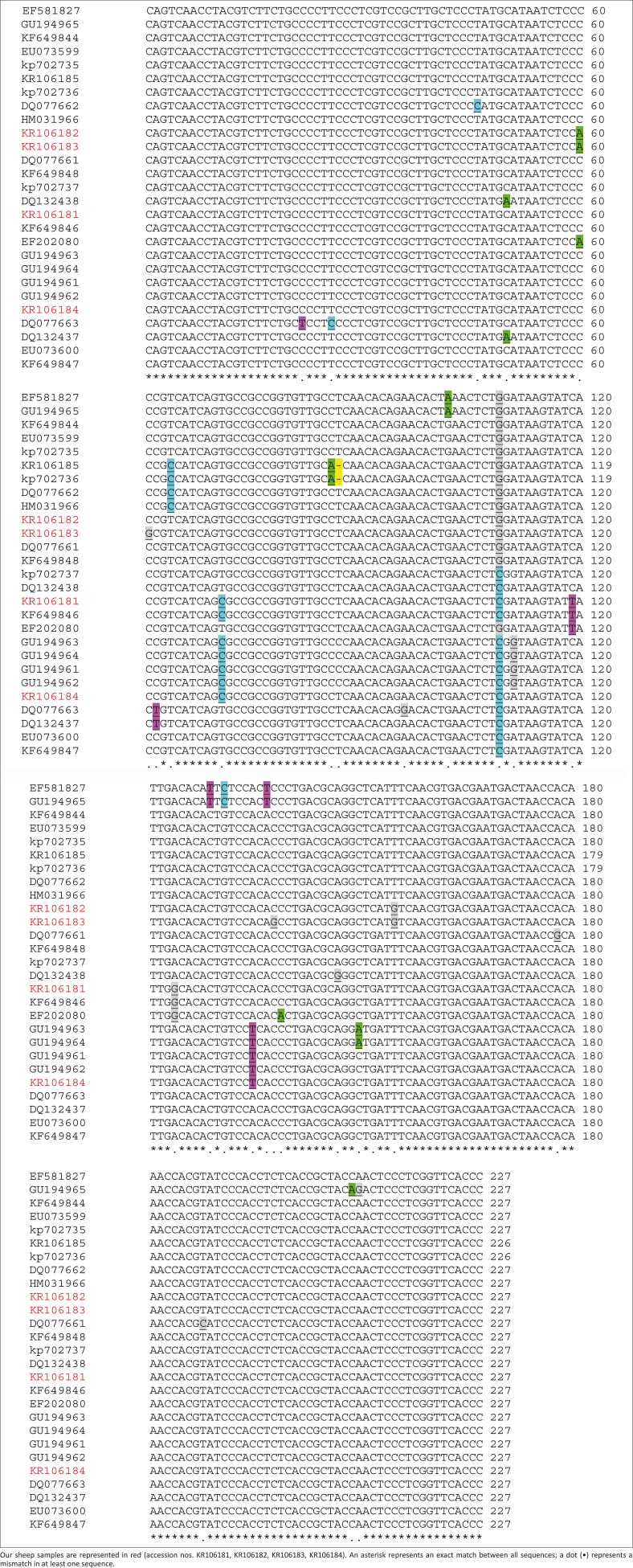
Sequence alignment of *Nc-5* gene of *Neospora caninum* from mammalian and bird hosts.
